# The wound healing effects of *Citrus latifolia* extracts: phytochemical profiling and in silico investigations

**DOI:** 10.1186/s12906-026-05309-2

**Published:** 2026-03-18

**Authors:** Mona T. M. Ghanem, Zeinab A. Elshahid, Wael M. Elsayed, Heba D. Hassanein, Shaimaa A. Gouhar

**Affiliations:** 1 Chemistry of Medicinal Plants Department, National Research Centre, Pharmaceutical Industry Research Institute, National Research Center, Dokki, Cairo, Egypt; 2 Chemistry of Natural and Microbial Products, Pharmaceutical Industry Research Institute, National Research Center, Dokki, Cairo, Egypt; 3 Medical Biochemistry Department, Medicine and Clinical Studies Research Institute, National Research Centre, Dokki, Cairo, Egypt

**Keywords:** *Citrus latifolia*, Dichloromethane, Gas chromatography, Cytotoxicity, Molecular simulation, Wound healing

## Abstract

**Background:**

*Citrus latifolia* is a natural source of biologically active compounds, particularly flavonoids and phenolic acids, which are known for their antioxidant and anti-inflammatory properties. These properties suggest a potential role in promoting tissue repair and wound healing.

**Method:**

Extracts and fractions of *C. latifolia* were phytochemically profiled and evaluated for antioxidant activity. The ethyl acetate (EtOAc) fraction was further assessed for wound healing potential using BJ-1 human skin fibroblast cells. In silico molecular docking and dynamics simulations were conducted to explore the interactions of representative compounds with wound-healing–related protein targets.

**Results:**

Twenty compounds were identified in sub-fraction 1, in which Citric acid tri-methyl ester (23%) and Oleic acid (7.57%) were considered the major compounds. Fourteen compounds were identified in sub-fraction 2; Methyl 7-hydroxyheptanoate (36.04%), 9-Hexadecenoic acid (12.29%) and 1, 8 cineole (6.92%) were the major constituents. Ten compounds were identified from the third fraction, in which 9-octadecenoic acid (63.43%) was the most significant, followed by Linalool (13.20%) and ρ- allyl anisole (5.24%). *Citrus latifolia* fruits showed total phenols (44.42 mg GAE/g) and total flavonoids (38.60 mg CE/g). The performance, interaction, and stability of the key chemicals discovered by GC–MS when bound to a protein's active site were predicted using a molecular dynamic simulation.

**Discussion:**

The significant results may be attributed to the major identified phenolic compounds mentioned above in EtOAc fractions, suggesting possible biological effects that should be investigated in the near future. Therefore, expanding this type of research is recommended to produce therapies for wound healing activity of natural origin with cheap, safe, and less harmful side effects.

**Supplementary Information:**

The online version contains supplementary material available at 10.1186/s12906-026-05309-2.

## Introduction

*Citrus latifolia* Tanaka is cultivated predominantly in tropical areas. Brazil is a significant producer. Acid limes are utilized as fresh fruit or juice, mostly as a blending agent to enhance beverage flavor [1]. Common names: Bearss lime, Tahiti lime, and Persian lime [2]. Nevertheless, in the mid-twentieth century, the utilization of medicinal plants diminished by twenty-five percent as researchers preferred the application of synthetic chemicals for disease treatment. The trend is shifting towards the preference for medicinal plants, which possess natural compounds that are effective, chemically balanced, and exhibit less adverse effects compared to manufactured chemicals [3].

The immune system's natural healing process restores tissue structure and function. Wound healing is a complex, multi-cellular process that prevents infection and regenerates tissue after injury. However, all wounds, especially non-healing ones, can negatively impact health, leading to immobility, distress, hospitalization, and increased morbidity and mortality. The process of wound healing is intricate and can be delineated into three distinct phases: inflammation, proliferation, and remodeling, ultimately restoring tissue integrity and strength [4]. Some *Citrus* species have wound healing activity such as *C. reticulata* [5] and *C. aurantifolia* Swigle [6].

Herbal bioactive chemicals have significantly influenced Herbal bioactive chemicals have significantly influenced medication development since their chemical identification in recent decades. Recent phytochemical studies, such as the characterization of *Curio radicans*, further emphasize how profiling secondary metabolites enhances our understanding of their therapeutic potential [7]. In the present era, a continuous influx of novel plant-derived agents contributes to expanding our therapeutic repertoire, such as vincristine, galantamine, and artemisinin. There is a steady increase in the quantity of novel herbal constituents that possess pharmacological activity, with a particular emphasis on anti-inflammatory chemicals [8].

Severe pathological alterations, such as scarring and delayed wound healing, can result from excessive inflammatory responses. Conversely, wound healing is frequently facilitated by well-managed inflammation [9, 10]. Therefore, finding a treatment plan that efficiently alters cytokines as well as immune-related cells at the place of damage is essential for wound healing to lower inflammation at the suitable times [11]. The genus *Citrus* (*Rutaceae*) encompasses numerous plant species that yield some of the most widely grown fruits globally, offering a significant amount of essential oils. In traditional medicine, these oils were utilized for their antitoxic, anti-inflammatory properties, antipyretic, sedative and cholagogue activity. The essential oil of *Citrus latifolia* Tanaka (CLEO) has several properties, including anti-inflammatory, antibacterial, diuretic, and eupeptic actions [12]. *Citrus* species possess anti-inflammatory properties that referred to the presences of active constituents in their essential oils, which decrease the production of inflammatory mediators and pro-inflammatory cytokines in RAW 264.7 cells treated with lipopolysaccharide [13]. Specifically, while some Citrus species have been studied for wound healing activity, no detailed phytochemical profiling combined with in vitro wound healing assays and molecular dynamics studies of *Citrus latifolia* extracts has been reported to date. Specifically, we selected PDGFRA (PDB ID: 5GRN) and VEGF-A (PDB ID: 1BJ1) as molecular targets due to their critical roles in wound healing. PDGFRA is involved in fibroblast proliferation and tissue regeneration, while VEGF-A promotes angiogenesis by stimulating endothelial cell proliferation and new blood vessel formation regeneration [14, 15]. Studying the interactions of *Citrus latifolia* phytochemicals with these proteins provides mechanistic insights into their wound healing activity and potential cytotoxicity profiles, as stable and specific interactions may indicate therapeutic efficacy with minimal off-target effects. Our study therefore addresses this gap by providing both experimental and computational insights into its wound healing potential. In our study, we evaluate the wound healing properties of polar fractions of *C.latifolia* fruit, in addition to phytochemical investigation of the Dichloromethane (DCM) and Ethyl acetate (EtOAc) fractions.

## Material and method

### Chemicals

3-(4,5-dimethylthiazolyl-2)−2,5-diphenyltetra-zolium bromide (MTT) was obtained from Sigma-Aldrich. Also, DMEM, fetal bovine serum, penicillin/streptomycin, DMEM-F12 medium and trypsin solution were purchased from Lonza Spain. All chemicals and other reagents used were of analytical grade.

### Extraction process

*Citrus latifolia* fruits were collected from the Egyptian local market in August 2022. Plant material was dried at room temperature and then ground to fine powder. About 400 g of the ground powdered fruit was coldly macerated in two liters of methanol (80%) (Sigma-Aldrich) and allowed to stand overnight for three days with occasional shaking; this process was repeated three times successively. Filtration was performed using Whatman filter paper No. 1(Wagtech Inter. England), This step is crucial for ensuring a clearer solution, allowing for more accurate analysis in subsequent experiments, then concentrated under pressure at 45 ᵒC temp using rotavapor. After drying, the crude extract (105 g) was successfully fractionated with increasing polarities solvents using a separating funnel. Three fractions were obtained: Petroleum ether (7 g), Dichloromethane (DCM) (8 g), and Ethyl acetate (18 g), respectively.

### GC/Mass chromatography of the DCM fractions

DCM fraction was dried (8 g) and subjected to Silica Gel Chromatography using a glass column with dimensions (100*4 cm) packed with silica gel (200 g) (70–230 mesh AST-Merck) eluted with Hexane/EtOAc with different ratios starting with hexane and increasing polarity gradually with DCM in which three fractions were collected. The fractions were analyzed by Gas Chromatography followed by a mass spectrometer for identification (Turbomass, PerkinElmer, Inc., Waltham, MA, USA), comparing the resulted masses to Adams and Wiley GC/MS Libraries by applying the same GC/MS conditions as [16].

### HPLC analysis of ethyl acetate (EtOAc) fraction

HPLC analysis was conducted utilizing an Agilent Technologies 1100 series liquid chromatograph, which was outfitted with an autosampler and a diode-array detector [17]. The mobile phase solvent part A was an acetonitrile-based mobile phase solvent, while Part B was a mixture of 2% CH_3_COOH (acetic acid) with water (v/v). An Eclipse XDB-C^18^ analytical column with a C^18^ guard column from Phenomenex in Torrance, CA, measuring 4.6 X 150 µm and 5 µm, was used. The gradient program was as follows: after 30 min, the flow rate was reduced to 85% B; after 20 min, it was reduced to 50% B; after 5 min, it was reduced to 0% B; and after 5 min, it was increased to 100% B. The flow rate was maintained at 0.8 ml/minute for the whole sixty-minute run. The benzoic acid and cinnamic acid derivatives were analyzed at 280 and 320 nm concurrently, while the flavonoids were examined at 360 nm, with an injection volume of 50 µl. A 0.45 µm Acrodisc syringe filter (Gelman Laboratory, MI) was used to filter all samples prior to injection. Using UV spectra and retention periods that were similar to the standards, we were able to identify the peaks.

### Qualification and quantification of total phenolic content of the ethyl acetate (EtOAc) extract

The total phenolic content assay was performed according to the Folin-Ciocalteu procedure [18]. The total content of phenolic was quantified using a calibration curve and expressed as milligrams of gallic acid equivalent (mg GAE) per gram of sample.

### Quantification and qualification of total flavonoid content of the ethyl acetate (EtOAc) extract

The total flavonoid content was assessed using the methodology of Žilić et al. [18] through an aluminum chloride (AlCl_3_) colorimetric assay. Briefly, three hundred microliters of 5% sodium nitrite (NaNO_2_) were mixed with one hundred microliters of extract. After six minutes, 300 μL of a 10% AlCl_3_ solution was added, and the total volume was adjusted to 2.5 mL with distilled water. Following a seven-minute interval, 1.5 mL of 1 M NaOH was added, and the resulting mixture underwent centrifugation at 5000 × g for ten minutes. The absorbance of the supernatant was quantified at 510 nm relative to the solvent blank. The total flavonoid content was quantified using a calibration curve established with catechin and represented as milligrams of catechin equivalent (mg C.E.) per gram of sample. If the observed absorbance value exceeded the linear range of the standard curve, further dilution was performed.

### Evaluation the radical scavenging activity for the ethyl acetate extract

According to Hwang and Do Thi [19] the free radical scavenging capacity for ethyl acetate extract was performed using the stable DPPH. The final concentration was 200 µM for DPPH, and the final reaction volume was three mL. The absorbance was measured at 517 nm against a blank of pure methanol after sixty minutes of incubation in a dark condition. The following equation calculated the percent inhibition of the DPPH free radical:

$$\begin{aligned}&\text{Inhibition }(\mathrm{\%})=\\&100\times [({\mathrm{A}}_{\mathrm{control}}-{\mathrm{A}}_{\mathrm{sample}}\mathrm{x})/{\mathrm{A}}_{\mathrm{control}}]\end{aligned}$$

where: A _sample_ means the absorbance with the test compound. A _control_ means the absorbance of the control reaction (containing all reagents except the test compound). The standard curve was prepared using Trolox. Results were expressed as mg Trolox equivalents. Additional dilution was needed if the DPPH value measured was over the linear range of the standard.

### Cell culture

The human dermal Fibroblast (BJ-1) normal cell line was cultured in DMEM-F12 medium supplemented with 10% fetal bovine serum at 37 °C in 5% CO_2_ and 95% humidity. Cells were subcultured using trypsin versene 0.15%. All experiments were conducted at the Bioassay-Cell Culture Laboratory, National Research Center, Cairo, Egypt. The cell line was kindly provided by Professor Stig Linder, Oncology and Pathology department, Karolinska Institute, Stockholm, Sweden, originally obtained from ATCC.

#### Cytotoxic activity on normal cells

Cytotoxicity of the prepared compounds (Catechin, P-Hydroxybenzoic acid and Protocatechuic acid) in BJ-1 normal cells was evaluated using a modified Thabrew et al*.* [20] method. Cells (20,000 cells/well) were incubated for 48 h in triplicate with various preparations at a final concentration of 100 µg/ml. Doxorubicin (100 µM) served as the positive control, while 0.5% DMSO was used as the negative control. Cell viability was determined via MTT assay following Mosmann [21]. Cytotoxicity was quantified using the formula:

$${\% cytotoxicity}=[1-(\text{AVx }/\text{ AVNC})] \times 100$$

where AVx represents the average absorbance of the sample wells and AVNC represents the average absorbance of the negative control wells, both measured at 595 nm with a 690 nm reference.

#### In vitro wound healing activity of different extracts

A modified scratch assay, based on the method described by Elshahid et al. [22] was employed to assess the migratory capacity of BJ-1 cells and the wound-healing potential of various preparations. BJ-1 cells (2 × 10^5^ cells/well) were seeded in 6-well plates and incubated in complete media at 37 °C and 5% CO₂. After 24 h, confluent monolayers were scraped with a sterile P200 pipette tip, and cellular debris was removed via PBS wash. Cells were then treated with the indicated preparations at 100 µg/mL, with untreated cells serving as the negative control. Initial (0 h) images of the induced scratch wounds were captured using phase-contrast microscopy at 40 × magnification. Following a 24-h incubation period, a second set of images was acquired. Wound closure was quantified using Image J software, and the percentage of wound closure was calculated as follows:

$$\begin{aligned}&\mathrm{Wound}\;\mathrm{closure}\;\left(\%\right)=\\&\left[\left(\mathrm{Wound}\;\mathrm{width}\;\mathrm{at}\;0\;\mathrm h-\mathrm{Wound}\;\mathrm{width}\;\mathrm{at}\;24\;\mathrm h\right)\right.\\&\left./\mathrm{Wound}\;\mathrm{width}\;\mathrm{at}\;0\;\mathrm h\right]\times100\end{aligned}$$

### Molecular docking studies

The X-ray crystallographic structures of the selected proteins, PDGFRA (PDB ID: 5GRN) and VEGF-A (PDB ID: 4QTB), were obtained from the Protein Data Bank (PDB). The UCSF Chimera (version 1.11.2) software was employed for protein structure preparation, where hydrogen atoms were added, and water molecules within 5 nm of the binding site were removed to enhance docking accuracy [23]. Subsequently, PyRx Python Prescription Virtual Screening Tool (version 0.8) was utilized for protein structure minimization using the AMBER force field, and grid boxes were generated at the active sites with a 20 Å radius around the crystallographic binding sites [24]. The 2D molecular structures of the synthesized compounds (ligands) were drawn using Chem-Draw Pro 8.0 and then subjected to 3D structure generation and geometry optimization using PyRx (version 0.8), followed by energy minimization via the AMBER force field to obtain its lowest-energy conformation [25]. The ligand preparation process involved hydrogen atom addition, salt removal, and ionization at pH 7 to ensure physiologically relevant conditions [26]. The docking process identified the optimal ligand–protein binding poses based on binding affinity scores and molecular interactions. The BIOVIA Discovery Studio Visualizer was employed to analyze and visualize the docked ligand–protein complexes, including identification of hydrogen bonds, hydrophobic interactions, and van der Waals contacts with specific amino acid residues in the binding site, as previously described [27, 28].

### Molecular dynamic simulations

To further validate the docking results, molecular dynamics (MD) simulations were conducted on protein–ligand complexes exhibiting the lowest binding energy. The simulations were performed using Nanoscale Molecular Dynamics (NAMD) software, ensuring a detailed exploration of the structural and dynamic behavior of the complexes [29]. Ligand topology files were generated based on the best-docked poses with the aid of the CHARMM-GUI web server and the NAMD module input generator. The CHARMM force field was applied to optimize ligand parameters [30]. Subsequently, the processed ligand files were incorporated into Visual Molecular Dynamics (VMD) software for further system preparation. The Protein Structure File (PSF) was generated, and both protein and ligand structures were pre-processed using custom VMD scripting [31]. To mimic a biological environment, the system was solvated using the solvate function in VMD. A 100 ns MD simulation was then executed to assess the stability of the protein–ligand complexes. Root mean square fluctuation (RMSF) was employed to evaluate the dynamic behavior of the complexes across the simulation trajectories, and the results were visualized using Gnu plot [32]. To estimate the binding affinities of the protein–ligand complexes, the MM-PBSA (Molecular Mechanics Poisson–Boltzmann Surface Area) approach was applied using the g_mmpbsa tool [33, 34].‏ Snapshots were extracted at 50 ps intervals from the last 10 ns of the MD trajectories. The total binding free energy (ΔG_bind) was calculated as:

$$\Delta {\mathrm{G}}_{\mathrm{bind}}={\mathrm{G}}_{\mathrm{complex}}-({\mathrm{G}}_{\mathrm{protein}}+{\mathrm{G}}_{\mathrm{ligand}})$$

where ΔG_bind was further decomposed into van der Waals (ΔE_vdW), electrostatic (ΔE_ele), polar solvation (ΔG_pol), and nonpolar solvation (ΔG_nonpol) contributions. Per-residue energy decomposition was also performed to identify key residues contributing to ligand binding. This approach has been successfully applied in previous studies for reliable estimation of ligand–protein interactions [33, 34].

## Results

### Phytochemical investigation of *C.latifolia* fractions

#### G.C./Mass analysis of dichloromethane extract fractions

GC/MS analysis of fraction 1 revealed the presence of 16 compounds (Table 1), which included Citric acid tri-methyl ester (43.06%), bergamotene (23.06%), and oleic acid (7.57%) were considered the major compounds. Twelve compounds were identified in fraction 2; the major compounds were Citric acid tri-methyl ester (36.04%), 9-hexadecenoic acid (12.29%), and 1,8 cineol (9.92%). Nine compounds were identified from the third fraction, 9-Octadecenoic acid (63.43%) being the most significant, followed by Linallol (13.20%).

**Table 1:**
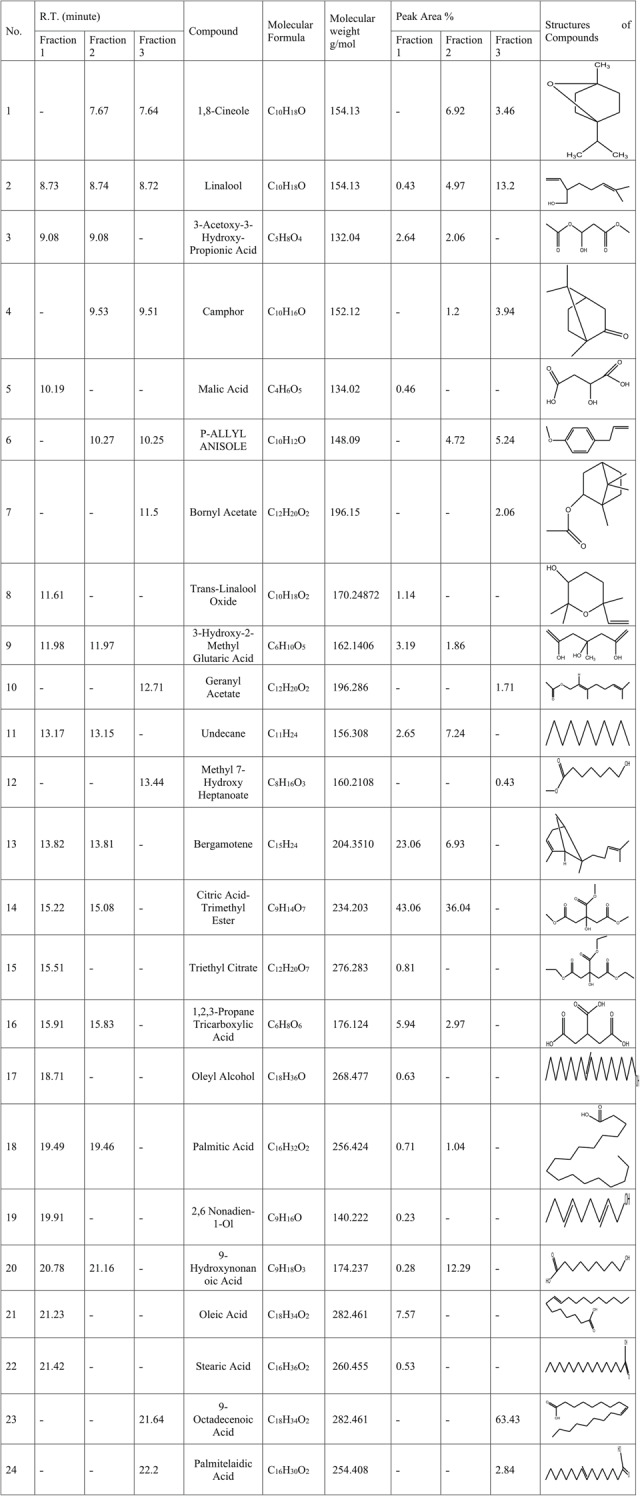
G.C./Mass analysis data of fractions 1,2 and 3 of DCM isolated from *C. latifolia* fruit

#### HPLC analysis of ethyl acetate fraction

In Table 2, Twenty phenolic and flavonoid compounds were identified by comparison to internal standards. The significant compounds found were Cateachin (2130.69 μg/gm), protocatechuic acid (1297.43 μg/gm), *p*-hydroxybenzoic (10,319.22 μg/gm) and Apigenin-7-glucoside (811.30 μg/gm).

**Table 2:** Phenolics and Flavonoids identified from the EtOAc fraction of *C.latifolia* applying HPLC analysis compared to standards

Compound	Retention time (min.)	Concentration (μg/gm)
Gallic	2.55	96.60
Protocatechuic	4.79	1297.43
Gentisic	5.83	320.07
*p*-hydroxybenzoic acid	7.17	10,319.22
Catechin	9.03	2130.69
Chlorogenic	9.43	179.19
Caffeic	10.01	56.59
Syringic	10.81	626.54
Vanillic	12.56	97.31
Ferulic	13.61	88.88
Sinapic	14.77	71.52
Rutin	-	ND
*p*-coumaric	15.29	95.75
Apigenin-7-glucoside	16.36	811.30
Rosmarinic	18.20	182.88
Cinnamic	19.20	207.31
Qurecetin	30.82	32.15
Apigenin	31.54	63.10
Kaempferol	32.80	558.14
Chrysin	-	ND

#### The measurement of ethyl acetate’s DPPH, total flavonoids, and total phenolics:

An investigation of the compositions of *C.latifolia* extract (EtOAc) is essential to justifying its cytotoxic activity. For quantitative determination, flavonoid content in the EtOAc extract is determined by colorimetric method using aluminum chloride (AlCl_3_). The results obtained from the calibration curve of catechin (0–120 mg/mL) and given in catechin equivalents (C.E.) per gram dry extract weight. In Table 3, the total flavonoid content in EtOAc fraction is 38.60 ± 0.53 mg C.E./g. Also, Phenolic content was determined using–Ciocalteu reagent. The results were obtained from the calibration curve of gallic acid (0–60 mg/mL) and given as gallic acid equivalents (GAE) per gram dry extract weight. The TPC in EtOAc extracts 44.42 ± 0.57 mg GAE/g as recorded in Table 3. Moreover, the evaluation of antioxidant activity was performed using DPPH assay. The results obtained were determined from a calibration curve using Trolox as standard solution and expressed as mg Trolox equivalent per gram dry extract weight for EtOAc was 68.49 ± 0.64 mg T.E./g that showed in Table 3.

**Table 3:** Total phenolics, total Flavonoids, and DPPH estimation

Fraction	T.P.C (mg GAE/gm)	T.F.C (mg CE/gm)	DPPH (mg TE/gm)
EtOAc	44.42 ± 0.57	38.60 ± 0.53	68.49 ± 0.64

^*^milligrams of catechin equivalent per g extract dry weight = mg CE/g, milligrams of gallic acid equivalent per g extract dry weight = mg GAE/g, milligrams of trolox equivalent per g extract dry weight = mg TE/g, T.P.C = total phenolic content, T.F.C = total flavonoid content

#### Cell viability by MTT assay

**Fig. 1:**
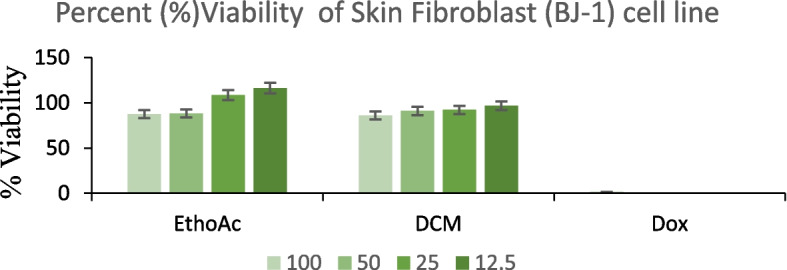
BJ-1 Cell Viability after treatment with different extracts Each value indicates the mean ± standard deviation and is representative of results obtained from three independent experiments

Figure 1 presents the viability of BJ-1 cells 48 h after treatment with the extracts at a concentration of 100 µg/mL. Data are expressed as mean ± standard deviation from three independent experiments, ensuring the reliability and reproducibility of the observed effects. The negative control (0.5% DMSO) exhibited high cell viability, as expected. Treatment with doxorubicin (100 µM), the positive control, significantly reduced cell viability, demonstrating its known cytotoxic effect. The EtOAc extract maintained high cell viability across all tested concentrations, with values above 90%. The highest concentration (100 µg/mL) showed a slightly lower viability but remained high, suggesting minimal cytotoxicity toward BJ-1 fibroblast cells. Similarly, the DCM extract-maintained cell viability above 80% across all concentrations, indicating no significant toxic effects on the skin fibroblasts.

#### Evaluation of in vitro wound healing activity of Ethyl acetate (EtOAc) and Dichloromethane

**Fig. 2:**
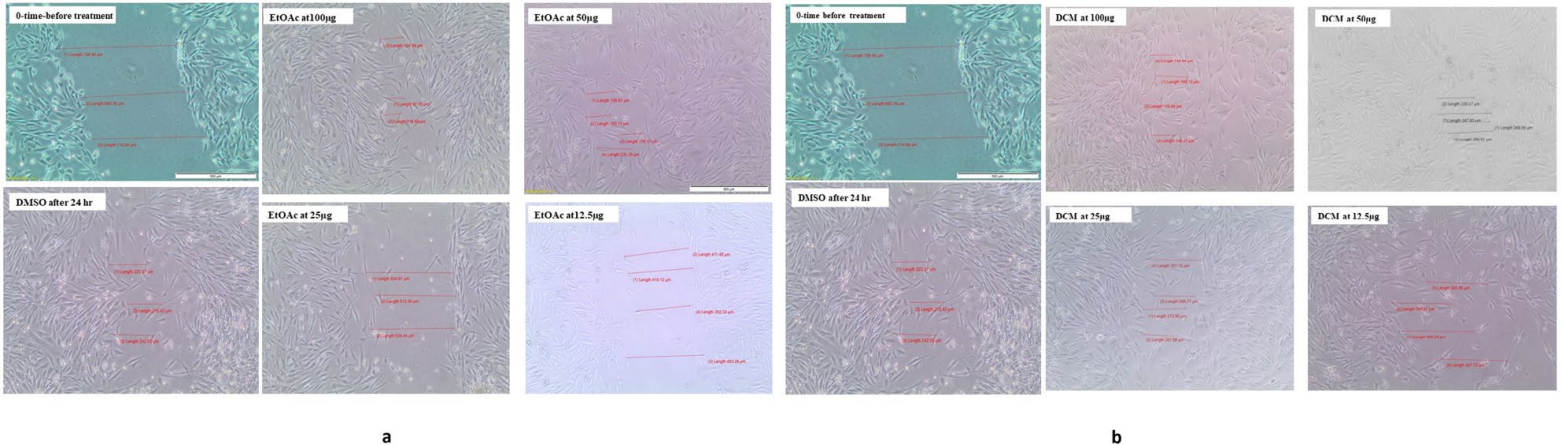
**a** Microscopical images representing the In vitro wound healing nature of different extracts on BJ-1 cells. Cells were treated with EtOAc extracts at four different concentrations (100, 50, 25 & 12.5) at 0 time. Images were acquired at 0-time and after 24hr. **b** Microscopical images representing the In vitro wound healing nature of different extracts on BJ-1 cells. Cells were treated with DCM extracts at four different concentrations (100, 50, 25 & 12.5) at 0 time. Images were acquired at 0-time and after 24hr. Images were analyzed using “image J” software, and percentage of the closed area was measured and compared with the value obtained at 0h

**Fig. 3:**
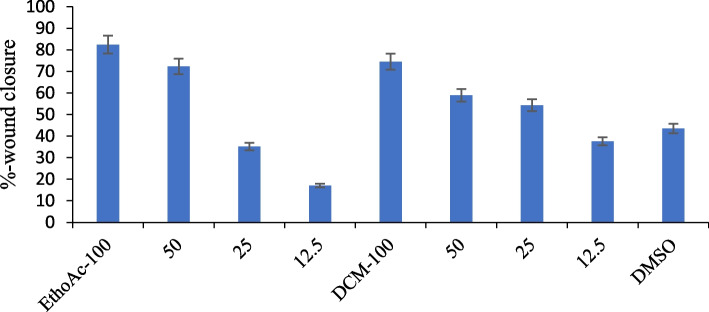
Wound healing ability of different extracts and DMSO on skin Fibroblast (BJ-1) normal cell line. The migration rates of BJ-1 cells were assessed by the scratch assay method. Cells were incubated with the different extracts and DMSO for 24 h at 37 C and 5% CO_2_. The scratch induced represented wound was photographed at 0 times and after 24 h. of incubation. The percentage of the closed area was measured and compared with the value obtained at 0 h. An increase in the percentage of the closed area indicated the migration of cells. Data were recorded in triplicate

The wound healing effect of different extracts was evaluated by measuring the percentage of wound closure at various concentrations. As shown in Figs. 2 & 3, the EtOAc extract at 100 µg/mL exhibited the highest wound closure percentage (82%), followed by 50 µg/mL (72%). Lower concentrations (25 and 12.5 µg/mL) showed significantly reduced closure, with 12.5 µg/mL showing the lowest activity (15%). The DCM extract at 100 µg/mL also promoted substantial wound closure (75%), followed by 50 µg/mL (58%). Lower concentrations showed decreased closure, with 12.5 µg/mL exhibiting minimal effect (30%). The vehicle control (DMSO) displayed moderate closure (~ 40%), representing baseline cell migration. Overall, higher concentrations of both extracts enhanced wound healing, with EtOAc showing slightly greater efficacy than DCM. Figure 2a & b: Representative images showing in vitro wound healing of BJ-1 cells treated with EtOAc and DCM extracts at 0 h and 24 h. Wound closure percentage was measured using ImageJ software.

### Molecular docking

**Fig. 4:**
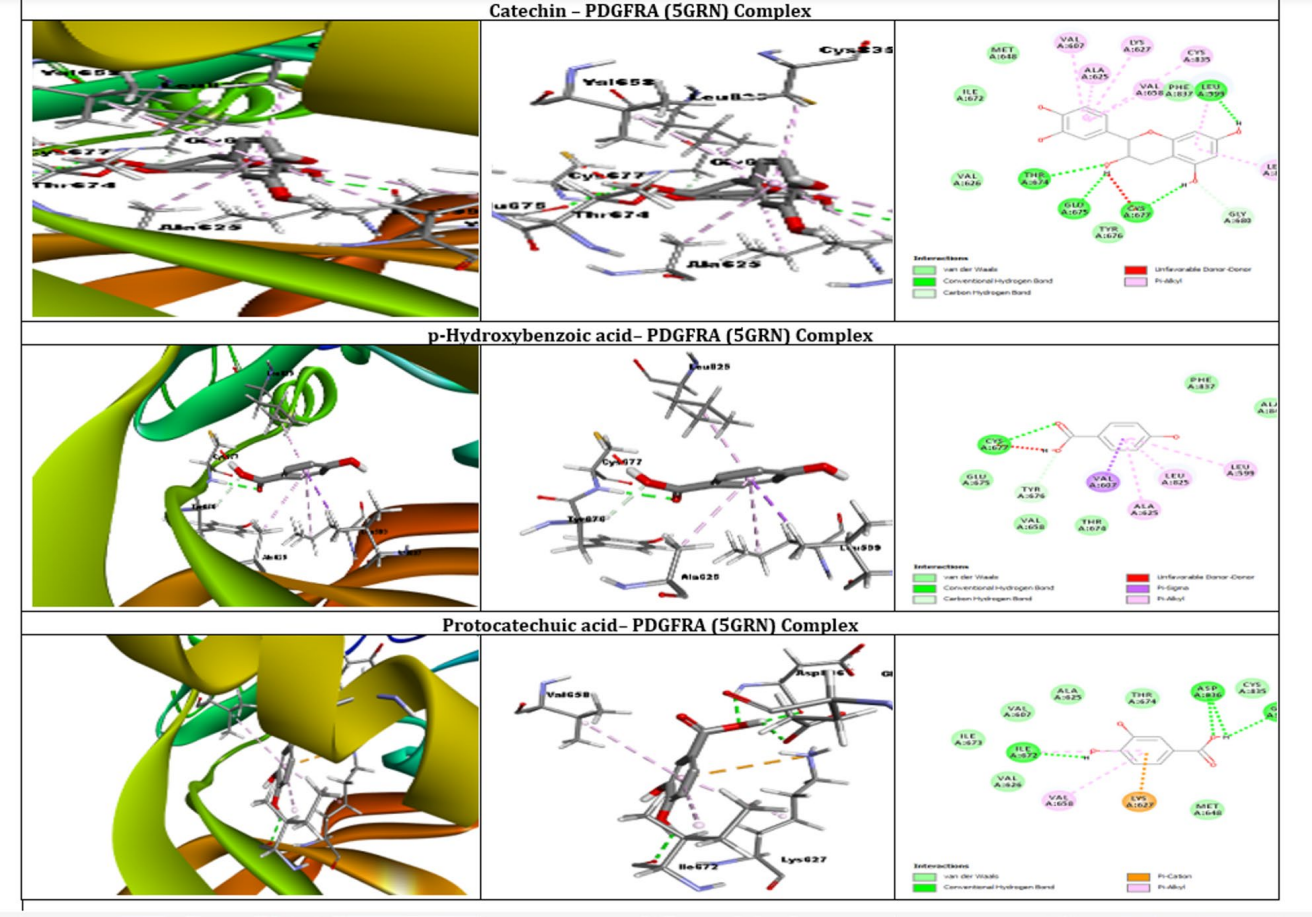
Molecular Docking Interactions of Catechin, p-Hydroxybenzoic Acid, and Protocatechuic Acid with PDGFRA (5GRN) Protein

**Fig. 5:**
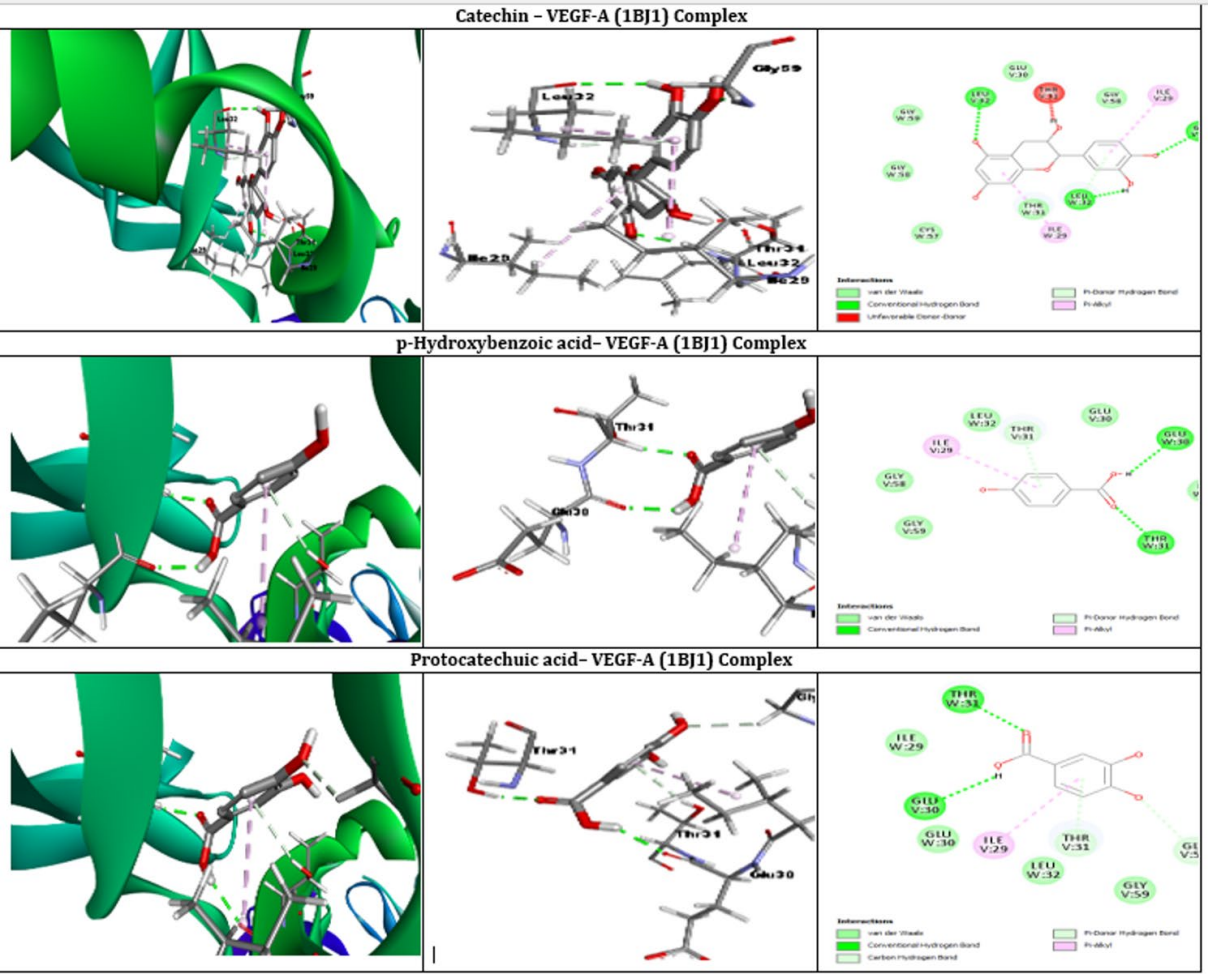
Molecular Docking Interactions of Catechin, p-Hydroxybenzoic Acid, and Protocatechuic Acid with VEGF-A (1BJ1) Protein

Molecular docking studies were performed to investigate the binding interactions of various ligands with the PDGFRA protein (PDB ID: 5GRN) and VEGF-A protein (PDB ID: 1BJ1). PDGFRA and VEGF-A were selected as molecular targets due to their critical roles in wound healing. VEGF-A regulates angiogenesis by promoting endothelial cell proliferation and new blood vessel formation, while PDGFRA contributes to fibroblast proliferation and tissue regeneration [35, 36]. Evaluating the interactions of *Citrus latifolia* phytochemicals with these proteins provides insight into the potential molecular mechanisms underlying the extract’s wound healing effects. Table 4 shows all ligand-receptor binding affinity and Tables 5 and 6 showed the best docked interactions between ligand and proteins, while Figs. (4) and (5) showed the interactions inside the binding site of the target, and the overall interactions of the promising docked compounds compared with the co-crystallized ligand.

**Table 4:** Binding affinities of ligands to PDGFRA and VEGF-A proteins (Kcal/mol)

Ligand	Binding Affinity with PDGFRA	Binding Affinity with VEGF-A
Catechin	−9.1	−7.6
P-Hydroxybenzoic acid	−5.8	−5.3
Protocatechuic acid	−4.9	−4.4
1,8-CINEOLE	−3.6	−3.5
9-hydroxynonanoic acid	−3.9	−3.1
9-octadecenoic acid	−4.2	−4.2
Bergamotene	−2.2	−3.3
Citric acid-trimethyl ester	−4.1	−4.1
Linalool	−3.7	−2.6

**Table 5:** Molecular Docking of the different ligands on PDGFRA (5GRN) protein

Ligands with PDGFRA	Binding affinity (Kcal/Mol)	Number of hydrogen bonds	Atoms forming hydrogen bonds	Other interactions	Amino acids involved in other interactions
Catechin	−9.1	2	THR674, GLU675	Van der Waals, Pi-Donor Hydrogen Bond, Pi-Alkyl	VAL626, ILE672, LEU599, LEU825, GLU607, LYS627, MET648, ALA625, VAL658, PHE837, CYS677, GLY680
*P*-Hydroxybenzoic Acid	−5.8	1	THR674	Van der Waals, Pi-Donor Hydrogen Bond, Pi-Alkyl	VAL626, ILE672, LEU599, LEU825, GLU607, LYS627, MET648, ALA625, VAL658, PHE837, CYS677, GLY680
Protocatechuic Acid	−4.9	2	THR674, GLU675	Van der Waals, Pi-Cation, Pi-Donor Hydrogen Bond, Pi-Alkyl	VAL626, ILE672, LEU599, LEU825, GLU607, LYS627, MET648, ALA625, VAL658, PHE837, CYS677, GLY680, ASP836

**Table 6:** Molecular Docking of the different ligands on VEGFA (1BJ1) protein

Ligands with VEGF-A protein	Binding affinity (Kcal/Mol)	Number of hydrogen bonds	Atoms forming hydrogen bonds	Other interactions	Amino acids involved in other interactions
Catechin	−7.6	2	THR31, THR31	van der Waals, Pi-Donor Hydrogen Bond, Pi-Alkyl	LEU32, ILE29, GLY58, GLY59, CYS57
*P*-Hydroxybenzoic Acid	−5.3	1	THR31	van der Waals, Pi-Donor Hydrogen Bond, Pi-Alkyl	LEU32, ILE29, GLY58, GLY59, GLU30
Protocatechuic Acid	−4.4	2	THR31, GLU30	van der Waals, Pi-Donor Hydrogen Bond, Pi-Alkyl	LEU32, ILE29, GLY58, GLY59

Regarding PDGFRA protein, Catechin exhibited the strongest binding affinity (−9.1 kcal/mol), forming two hydrogen bonds with THR674 and GLU675 and engaging in multiple non-covalent interactions (van der Waals, Pi-Donor Hydrogen bonds, Pi-Alkyl) with residues VAL626, ILE672, LEU599, LEU825, GLU607, LYS627, MET648, ALA625, VAL658, PHE837, CYS677, and GLY680. Protocatechuic acid, with a binding affinity of −4.9 kcal/mol, also formed two hydrogen bonds (with THR674 and GLU675) and exhibited van der Waals interactions, Pi-Donor Hydrogen bonds, Pi-Alkyl interactions, and Pi-Cation interactions with residues VAL626, ILE672, LEU599, LEU825, GLU607, LYS627, MET648, ALA625, VAL658, PHE837, CYS677, GLY680, and ASP836. While P-hydroxybenzoic acid, with a binding affinity of −5.8 kcal/mol, formed a single hydrogen bond with THR674 and interacted through van der Waals forces, Pi-Donor Hydrogen bonds, and Pi-Alkyl interactions with residues VAL626, ILE672, LEU599, LEU825, GLU607, LYS627, MET648, ALA625, VAL658, PHE837, CYS677, and GLY680.

Molecular docking studies with the VEGF-A protein revealed binding affinities of −7.6, −5.3, and −4.4 kcal/mol for catechin, P-hydroxybenzoic acid, and protocatechuic acid, respectively. Catechin exhibited two hydrogen bonds, specifically with the oxygen atoms of THR31. Additionally, various non-covalent interactions, including van der Waals forces, Pi-Donor Hydrogen bonds, and Pi-Alkyl interactions, were observed with residues LEU32, ILE29, GLY58, GLY59, and CYS57. P-Hydroxybenzoic acid displayed a single hydrogen bond with the oxygen atom of THR31, along with van der Waals forces, Pi-Donor Hydrogen bonds, and Pi-Alkyl interactions with residues LEU32, ILE29, GLY58, GLY59, and GLU30. Protocatechuic acid formed two hydrogen bonds, one with THR31 and another with GLU30, and exhibited van der Waals forces, Pi-Donor Hydrogen bonds, and Pi-Alkyl interactions with residues LEU32, ILE29, GLY58, and GLY59. The remaining ligands (1,8-cineole, 9-hydroxynonanoic acid, 9-octadecenoic acid, bergamotene, citric acid-trimethyl ester, and linalool) showed lower binding affinities, indicating weaker interactions with the selected proteins.

### Molecular dynamics (MD) simulation

**Fig. 6:**
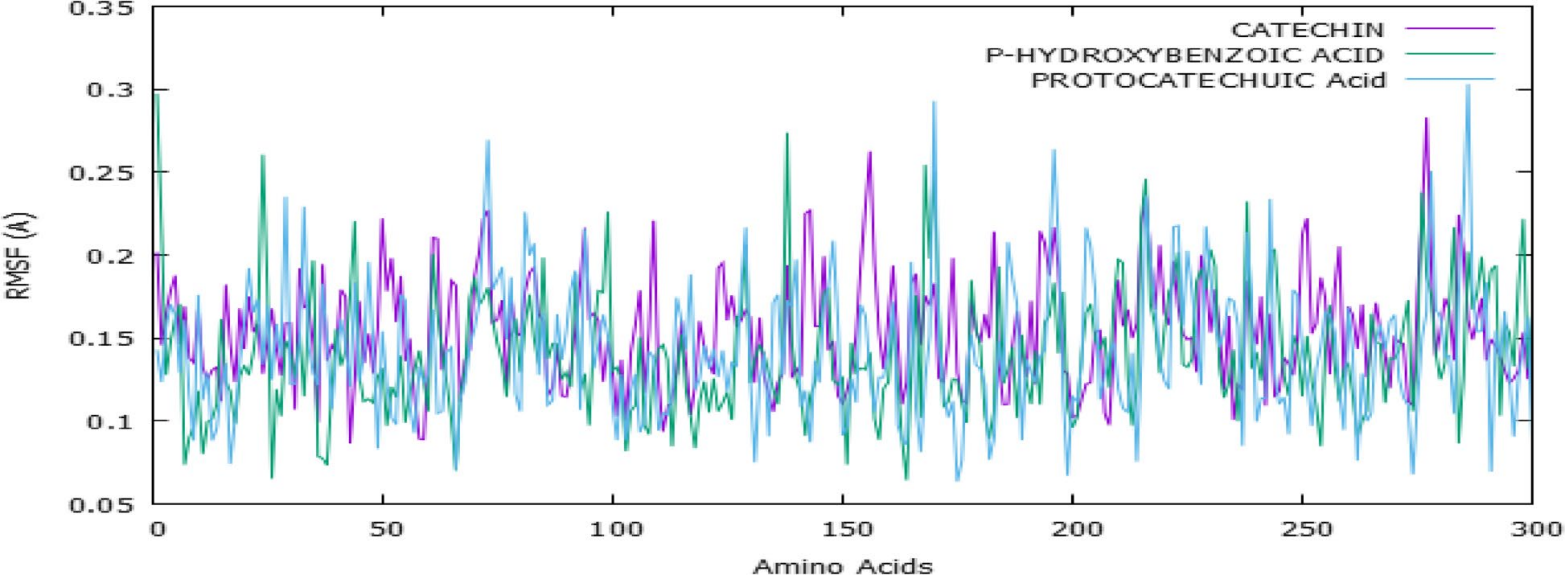
Root Mean Square Fluctuation (RMSF) analysis of PDGFRA protein complexed with catechin, p-hydroxybenzoic acid, and protocatechuic acid over a 100 ns MD simulation

**Fig. 7:**
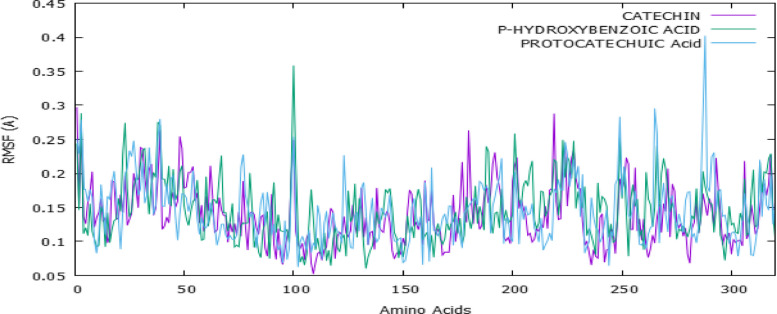
Root Mean Square Fluctuation (RMSF) analysis of VEGF-A protein complexed with catechin, p-hydroxybenzoic acid, and protocatechuic acid over a 100 ns MD simulation

To evaluate the conformational stability and flexibility of the protein–ligand complexes, we performed Root Mean Square Fluctuation (RMSF) analysis on the Cα atoms of all residues over a 100 ns MD simulation. As shown in Fig. 6, the RMSF values for all residues with PDGFRA of VEGF-A protein remained within the range of 0.05 Å to 0.35 Å, indicating overall stability throughout the simulation period. The three selected compounds [catechin (purple), P-hydroxybenzoic acid (green), and protocatechuic acid (blue)]; exhibited similar fluctuation trends, suggesting that their binding did not cause significant structural perturbations in the protein. Residues within the binding pocket showed minimal fluctuations, highlighting stable interactions between the protein and ligands. In case of VEGFA protein, as illustrated in Fig. 7, minor peaks were observed, likely corresponding to loop regions and flexible termini, residues in the binding pocket displayed minimal fluctuations, further supporting the stability of protein–ligand interactions. The MM-PBSA calculations provided a more accurate evaluation of binding affinities than the initial docking scores, as they incorporate solvation and entropic effects [33, 34]. Among the tested ligands, catechin exhibited the strongest binding, with a predicted ΔG_bind of approximately –45 kcal/mol.

p-Hydroxybenzoic acid and protocatechuic acid displayed moderate binding affinities, with ΔG_bind values –20 kcal/mol. Non-phenolic compounds, such as 1,8-cineole and citric acid trimethyl ester, showed weak and transient interactions, with ΔG_bind values greater than –10 kcal/mol, indicating poor binding stability.

## Discussion

The discovery and development of new therapeutic agents are crucial in advancing treatment strategies. It is essential to prioritize the use of safe compounds to minimize cytotoxicity and adverse effects on normal tissues. Natural compounds with low toxicity profiles offer promising alternatives without causing inflammation or tissue damage [37]. We started our study with investigating the effect of the extracts on Cell Viability of normal human fibroblast (BJ-1) cells at the tested concentrations**.** The most significant observation in this assay is the high degree of cell viability observed across all concentrations of both EtOAc and DCM extracts. Even at the highest concentration, cell viability remained above 90%, indicating minimal cytotoxicity. This suggests that these extracts, at least within the tested concentration range, are relatively safe for BJ-1 cells. The low cytotoxicity observed in BJ-1 cells has important implications for the safety profile of these extracts. This encouraged us to explore if these extracts could be used for potential therapeutic applications, such as wound healing. The wound healing activity of the studied extracts was then explored to achieve this aim.

Based on our results, EtOAc at 100 µg/mL appears to have the best wound healing activity. It shows the highest percentage of wound closure among all the tested extracts and concentrations. The results suggest that EtOAc possesses wound-healing potential, as evidenced by its ability to enhance wound closure in BJ-1 cells, especially at higher concentrations. This effect is likely due to the presence of bioactive compounds in the extract that promote cell migration and proliferation, the key processes involved in wound healing. The concentration-dependent effect suggests that higher concentrations deliver a greater amount of these active compounds, leading to a more pronounced effect.

In contrast, the DCM extract exhibited a more complex, possibly biphasic effect. At lower concentrations, DCM appeared to inhibit wound closure, which may be attributed to the activity of certain inhibitory compounds that dominate at these levels. However, at higher concentrations (100 µg/mL), these inhibitory effects were less evident, likely due to the presence or higher relative abundance of other compounds with positive wound-healing activity that counterbalance the inhibition. Such non-linear responses are not uncommon in phytochemical mixtures, where the biological effect reflects the combined and sometimes opposing actions of multiple constituents [38]. These differences between EtOAc and DCM highlight the importance of the extraction solvent, as each solvent yields distinct phytochemical profiles that contribute to varying biological activities [39].

Identifying compounds that can selectively modulate key proteins involved in wound healing, while maintaining safety, is crucial for developing effective and clinically viable wound-healing therapies. In this study, molecular docking was employed to investigate the molecular mechanism underlying the wound-healing activity of the selected extracts. By targeting key proteins involved in wound repair, such as VEGF-A or PDGFRA, we aimed to identify potential interactions that contribute to tissue regeneration and wound healing effects. The docking analysis allowed us to predict the binding affinities and interactions between bioactive compounds from the extracts and these critical proteins, providing insights into their possible role in modulating cellular processes essential for wound healing [40]. PDGFRA and VEGF-A are critical proteins involved in wound healing, primarily through their roles in cell proliferation, angiogenesis, and tissue regeneration [41, 42]. PDGFRA, a tyrosine kinase receptor, is activated by platelet-derived growth factors (PDGFs), promoting the recruitment and proliferation of fibroblasts, which are essential for extracellular matrix formation and wound contraction [43]. VEGF-A, a key regulator of angiogenesis, stimulates the migration and proliferation of endothelial cells, enhancing blood vessel formation to ensure adequate oxygen and nutrient supply to the healing tissue [44]. The interplay between PDGFRA and VEGF-A is crucial in orchestrating an efficient wound healing response.

The molecular docking studies revealed varying binding affinities of the tested ligands with the PDGFRA and VEGF-A proteins. Catechin exhibited the highest binding affinity with both proteins, suggesting the strongest interaction. This is likely due to the presence of multiple hydrogen bonds and other favorable interactions with key amino acid residues within the binding site. The presence of hydrogen bonds, van der waals forces, pi-donor Hydrogen bonds, pi-alkyl interactions, and Pi-Cation interactions plays a crucial role in ligand–protein binding. These interactions contribute to the overall stability and specificity of the ligand–protein complex [45]. The results suggest that catechin has the highest potential for binding to and interacting with the PDGFRA protein among the tested ligands. Comparable results were reported in *Euphorbia parviflora*, where phenolic compounds such as catechin were identified as dominant constituents and linked with antioxidant and protective properties [46]This supports our docking and MD findings on the relevance of phenolics to wound-healing mechanisms.”

To validate docking results and evaluate the conformational stability and flexibility of the protein–ligand complexes, we performed RMSF analysis on the Cα atoms of all residues over a 100 ns MD simulation. RMSF provides insights into the structural flexibility of the protein and helps determine the effect of ligand binding on protein dynamics [47]. The RMSF analysis suggests that all three ligands maintain strong and stable interactions within PDGFRA, as indicated by the low RMSF values (< 0.4 Å) across most residues. The minimal fluctuations in binding site residues confirm that the ligands effectively stabilized the protein structure, preventing significant conformational rearrangements during the simulation. Overall, these findings reinforce the binding efficiency and stability of the selected ligands, supporting their potential as effective PDGFRA regulators.

The MM-PBSA results provide deeper insights into the molecular basis of ligand binding, complementing and refining the initial docking predictions. Catechin emerged as the most potent binder, reflecting a combination of strong hydrogen bonding and extensive hydrophobic interactions that likely contribute to its high stability within the binding pocket. In contrast, p-hydroxybenzoic acid and protocatechuic acid showed moderate binding affinities, which can be attributed to a reduced number of stabilizing interactions. The weak binding observed for non-phenolic compounds such as 1,8-cineole and citric acid trimethyl ester indicates that these molecules are less compatible with the active site environment, consistent with their minimal hydrogen bonding and hydrophobic contacts. Overall, these findings highlight the utility of MM-PBSA in capturing solvation and entropic contributions to binding free energy, offering a more realistic assessment of ligand efficacy than docking alone. Moreover, the per-residue energy decomposition underscores specific amino acid residues that contribute most significantly to ligand stabilization, providing valuable guidance for future structure-based optimization of bioactive compounds. These observations are in agreement with previous studies emphasizing MM-PBSA as a reliable approach for ranking ligands and predicting binding stability [33, 34].

Comparative studies have reported similar findings with structurally related polyphenolic compounds. For example, Sari et al*.* [48] demonstrated strong binding affinities of flavonoids against receptor tyrosine kinases, reinforcing the role of hydrogen bonding and hydrophobic contacts in stable interactions. Similarly, Sari et al*.* [14] highlighted catechin and related phenolics as potent stabilizers of angiogenesis-related proteins, consistent with our observations for VEGF-A. Moreover, [15] reported that phenolic acids such as protocatechuic acid exert moderate yet biologically relevant binding interactions with signaling proteins, which aligns well with our docking and MM-PBSA results. Taken together, these comparisons strengthen the validity of our findings and highlight the pharmacological potential of *Citrus latifolia* phytochemicals in wound-healing contexts. Nevertheless, the absence of in vivo wound models represents a limitation of the current work. This study was intentionally designed as a preliminary in vitro evaluation to establish safety and activity before moving into animal studies. Ethical and financial considerations restricted us from conducting animal experiments at this stage. Future research will address this gap by validating the wound healing efficacy of *Citrus latifolia* extracts in appropriate animal models.

## Conclusion

This study demonstrates that *Citrus latifolia* EtOAc and DCM extracts possess significant wound-healing potential, with the EtOAc fraction showing the most pronounced effect by promoting fibroblast migration and wound closure while maintaining excellent cell viability. These findings confirm the safety of the extracts at the tested concentrations and support their potential as non-toxic, natural wound-healing agents. Molecular docking and molecular dynamics simulations further revealed stable and favorable interactions of catechin, p-hydroxybenzoic acid, and protocatechuic acid with VEGF-A and PDGFRA, key regulators of angiogenesis and fibroblast proliferation. By targeting these pathways, the extracts may accelerate tissue regeneration, vascularization, and overall repair processes. Collectively, this integrative experimental and computational approach provides strong evidence for the therapeutic promise of *C. latifolia* phytochemicals and lays a foundation for future in vivo studies and potential development of safe, affordable, and effective wound-healing formulations of natural origin.

## Supplementary Information

Supplementary Material 1.

## Data Availability

The data and materials are available from the corresponding author upon request.
