# Correction: The wound healing effects of *Citrus latifolia* extracts: phytochemical profiling and in silico investigations

**DOI:** 10.1186/s12906-026-05408-0

**Published:** 2026-06-17

**Authors:** Mona T. M. Ghanem, Zeinab A. Elshahid, Wael M. Elsayed, Heba D. Hassanein, Shaimaa A. Gouhar

**Affiliations:** 1 Chemistry of Medicinal Plants Department, Pharmaceutical Industry Research Institute, National Research Center, Dokki, Cairo Egypt; 2 Chemistry of Natural and Microbial Products, Pharmaceutical Industry Research Institute, National Research Center, Dokki, Cairo Egypt; 3 Medical Biochemistry Department, Medicine and Clinical Studies Research Institute, National Research Centre, Dokki, Cairo Egypt

**Correction: BMC Complement Med Ther 26, 129 (2026)**

**https://doi.org/10.1186/s12906-026-05309-2**

Following publication of the original article [1], the authors identified an error in the author names of Mona T. M. Ghanem and Heba D. Hassanein.

The incorrect author names are: Mona T. M. Ghanema and Heba D. Hassaneina.

The correct author names are: Mona T. M. Ghanem and Heba D. Hassanein.

The author group has been updated above and the original article [1] has been corrected.
