# Supplementary material for: The wound healing effects of Citrus latifolia extracts: phytochemical profiling and in silico investigations
Source: BMC Complement Med Ther. 2026 Mar 18;26:129. doi: 10.1186/s12906-026-05309-2 (PMC13063849; doi:10.1186/s12906-026-05309-2)
Supplement: Supplementary file 1 — Supplementary Material 1. [file 12906_2026_5309_MOESM1_ESM.doc]

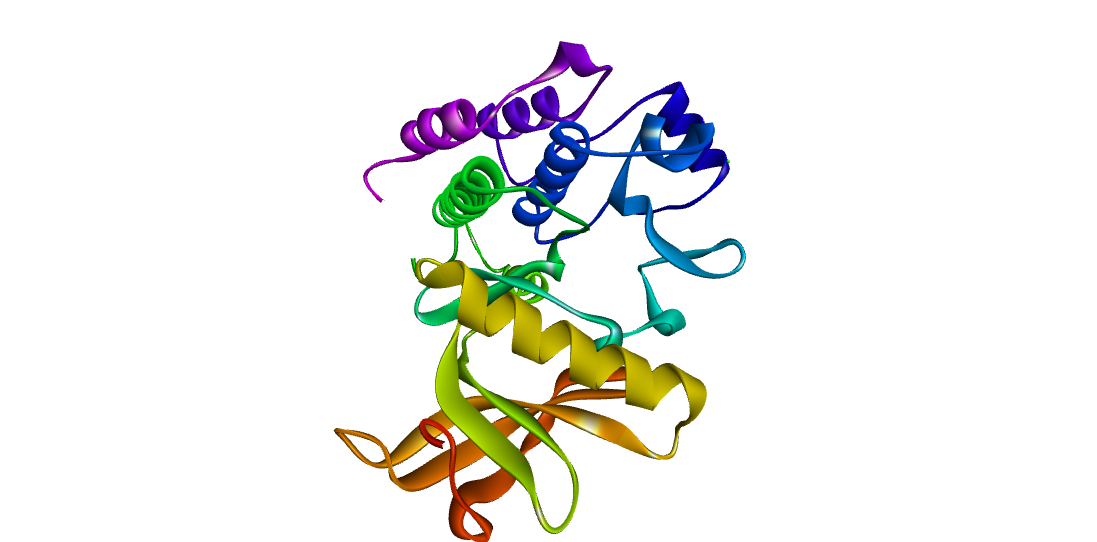


**PDGFRA (5GRN)**


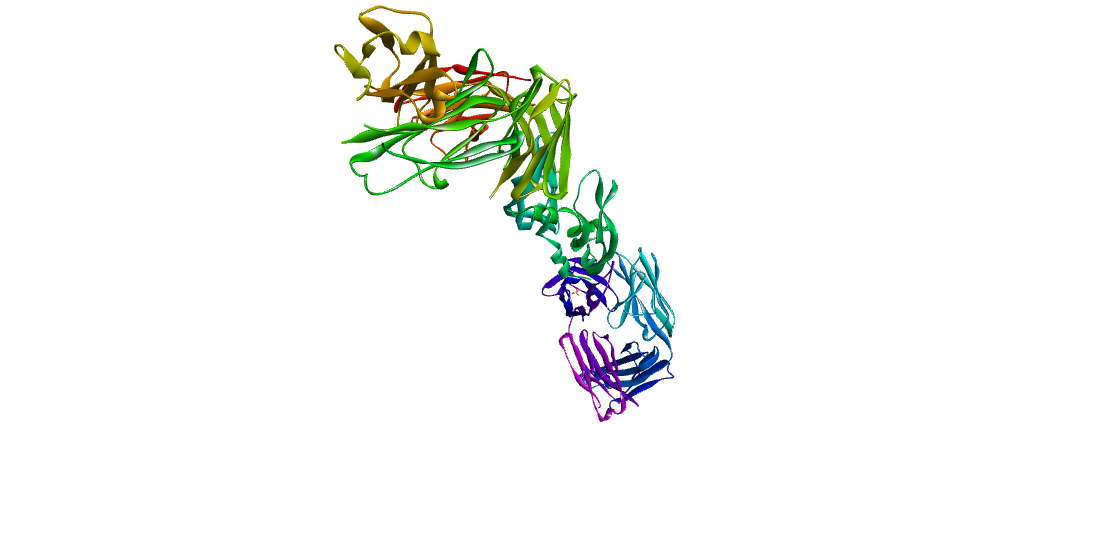


**VEGF-A (1BJ1)**

**Figure S1:** Graphical representation of the crystallographic structures of PDGFRA, VEGF
